# Predicting the O’Kelly-Marotta scale score after flow-diverter stent placement using silent MRA

**DOI:** 10.1007/s11604-024-01632-1

**Published:** 2024-08-29

**Authors:** Aki Miyazaki, Mizuho Nishio, Atsushi Fujita, Masaaki Kohta, Yasuyuki Kojita, Shintaro Horii, Takashi Sasayama, Takamichi Murakami

**Affiliations:** 1 Department of Radiology, Kobe University Graduate School of Medicine, 7-5-2 Kusunoki-Cho, Chuo-Ku, Kobe, 650-0017 Japan; 2 Department of Neurosurgery, Kobe University Graduate School of Medicine, 7-5-2 Kusunoki-Cho, Chuo-Ku, Kobe, 650-0017 Japan; 3 Center for Radiology and Radiation Oncology, Kobe University Hospital, 7-5-2 Kusunoki-Cho, Chuo-Ku, Kobe, 650-0017 Japan

**Keywords:** Flow-diverter, DSA, O'Kelly-Marotta grading scale, MRA, Silent MRA

## Abstract

**Purpose:**

Flow-diverter (FD) stents were developed to treat aneurysms that are difficult to treat with conventional coiling or surgery. This study aimed to compare usefulness of Silent MRA and TOF (time of flight) -MRA in patients with aneurysms after FD placement.

**Materials and methods:**

We retrospectively collected images from 22 patients with 23 internal carotid artery aneurysms treated with FD. Two radiologists conducted MRA and DSA experiments. In the first reading experiment, the radiologists evaluated the aneurysm filling by employing Silent MRA and TOF-MRA and utilizing the modified O’Kelly-Marotta (OKM) scale, a four-class classification system for aneurysms after FD placement. We then calculated the agreement between the modified OKM scale on MRA and the original OKM scale on DSA. In the second reading experiment, the radiologists rated blood flow within the FD using a five-point scale.

**Results:**

The weighted kappa value of the OKM scale between DSA and TOF-MRA was 0.436 (moderate agreement), and that between DSA and Silent MRA was 0.943 (almost perfect agreement). The accuracies for the four-class classification were 0.435 and 0.870 for TOF-MRA and Silent MRA, respectively. The mean score of blood flow within FD for TOF-MRA was 2.43 ± 0.90 and that for Silent MRA was 3.04 ± 1.02 (*P* < 0.001).

**Conclusion:**

Silent MRA showed a higher degree of agreement than TOF-MRA in aneurysm filling with DSA. In addition, Silent MRA was significantly superior to TOF-MRA in depicting blood flow within the FD. Therefore, Silent MRA is clinically useful for the follow-up of patients after FD placement.

**Supplementary Information:**

The online version contains supplementary material available at 10.1007/s11604-024-01632-1.

## Introduction

A flow-diverter (FD) stent is a device developed for the treatment of aneurysms that are difficult to treat with conventional coiling or surgery. A prospective study has demonstrated the treatment of uncoilable or failed internal carotid aneurysms with FD [1, 2]. As a result of this trial, FD placement has been considered safe and effective for uncoilable or failed aneurysms, contributing to its widespread use. A large aneurysm in the internal carotid siphon is a typical indication for FD [3]. Instead of the conventional treatment for completely blocked aneurysms, FD placement reduces blood flow within the aneurysm by placing a dense stent and promoting thrombosis within the aneurysm over several months or years [4].

Patients who undergo FD placement require long-term follow-up. In a report of 445 cases, complete occlusion was achieved in 72%, 78%, and 87% of cases at 6 months, 1 year, and 2 years, respectively [5]. DSA is considered to have the highest accuracy for evaluating aneurysm filling and blood flow in stents.

The O’Kelly-Marotta (OKM) grading scale is used to assess aneurysms treated with flow diversion by evaluating aneurysm filling and angiographic phases on DSA [6, 7]. The OKM scale assigns aneurysm filling to four classes: A (aneurysm filling, > 95%); B (aneurysm filling, 5–95%); C (aneurysm filling, < 5%); or D (aneurysm filling, 0%).

However, DSA is an invasive examination that requires hospitalization and is associated with a high risk of allergy or nephrotoxicity due to contrast media. CTA also has a relatively high accuracy; however, it also causes allergy or nephrotoxicity due to contrast media. Additionally, CTA is unsuitable for patients treated with both FD and additional coiling because of metal artifacts of the coils. Non-contrast MRA is considered suitable for repeated follow-up because it is non-invasive compared to DSA or CTA [8].

Silent MRA [9–14] can create images without a high thrombus signal because it is created from arterial spin labeling [15, 16], which is developed by subtracting the control image from the image obtained by the labeling pulse. However, a strong thrombus signal is not inevitable on time of flight (TOF)-MRA [17]. Furthermore, using the technology of ultra-short echo time makes it possible to reduce metal artifacts. Silent MRA can complement the weaknesses of conventional TOF-MRA and is gaining attention. Silent MRA is considered clinically effective for patients with aneurysms after FD placement if it is possible to predict the OKM scale using Silent MRA.

This study aimed to compare the usefulness of Silent MRA and TOF-MRA in patients with aneurysms after FD placement. For this purpose, we proposed our modified OKM scale, in which a four-class classification was performed to compare the aneurysm filling more precisely in Silent MRA and TOF-MRA. In a previous study, Oishi et al. evaluated the usefulness of Silent MRA and TOF-MRA after FD placement [18]. While they graded the aneurysm filling using a two-class classification (complete occlusion and incomplete occlusion), we used a four-class classification in this study. Furthermore, the imaging time for Silent MRA in our study (approximately 7 min) was shorter than the 12 min reported in a previous study by Oishi et al. In our study, the imaging time was adjusted to ensure that there was no major difference in the imaging time between Silent MRA and TOF-MRA, and to keep the imaging time of Silent MRA clinically acceptable. We also evaluated the agreement rate between the readers in the MRA evaluation.

## Materials and methods

This study was approved by the Ethics Review Board of ANONYMIZED (Approval number: ANONYMIZED). According to the Ethics Review Board, informed consent was not required because this study was non-invasive and retrospective. This study conformed to the Declaration of Helsinki and the Ethical Guidelines for Medical and Health Research Involving Human Subjects in Japan (https://www.mhlw.go.jp/file/06-Seisakujouhou-10600000-Daijinkanboukouseikagakuka/0000080278.pdf).

## Patient and aneurysm characteristics

**Fig. 1:**
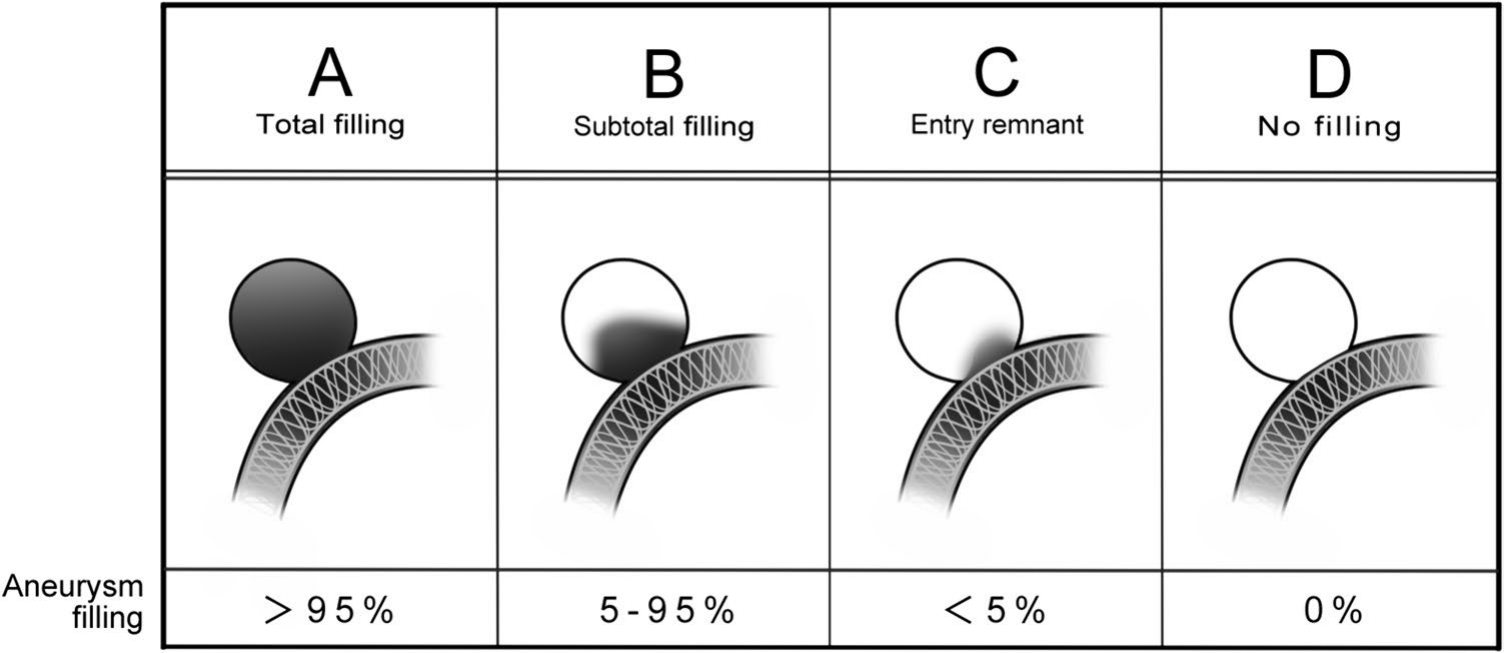
Schematic illustration of modified O’Kelly-Marotta scale

We retrospectively collected images of patients treated with FD for internal carotid artery (ICA) aneurysms at one institution between January 2021 and July 2022. DSA, TOF-MRA, and Silent MRA were performed approximately 6 months after FD placement. During this period, 25 patients with 26 aneurysms were treated, one of whom had a bilateral ICA aneurysm. Silent MRA was not available for two patients. In addition, one patient was excluded because follow-up DSA could not be performed due to renal dysfunction. Finally, 22 patients with 23 aneurysms were included in this study (Fig. 1). The FDs used in this study were a pipeline embolization device ([PED]; Medtronic, Dublin, Ireland) [3] or a flow-direction endoluminal device ([FRED]; Microvention, Tustin, California, USA) [19, 20].

Patient characteristics are summarized in Table 1. There were no deaths during treatment or follow-up and no cases of serious complications, such as aneurysm rupture, bleeding, or stent occlusion. PED was used in 20 cases and FRED in three cases. Among them, two FDs were used in one case. The FD and the two types of coils were used in the three cases of FRED. After FD placement, patients received aspirin and cilostazol for thromboprophylaxis.

**Table 1:** Characteristics of the patients treated with flow-diverter devices

Item for patient and aneurysm	Mean ± SD or ratio
Age	70.7 ± 14.3(47–85)
Sex (M/F)	6:17
Aneurysm size (mm)	15.6 ± 12.4
Symptom ( ±)	16:7
Location (cavernous segment/paraclinoid segment)	14:9
FD (PED/FRED)	20:3
Additional coiling ( ±)	4:19
Complication	0

*FD *flow-diverter, *FRED *flow-direction endoluminal device, *PED* pipeline embolization device, *SD* standard deviation

## Imaging parameters

TOF-MRA and Silent MRA were performed using a 3 T MRI (SIGNA PREMIER; GE Healthcare, United States). Imaging parameters for Silent MRA were as follows: TR/TE, 599/0.016 ms; flip angle, 5°; FOV, 180 × 180 mm; matrix, 160 × 160; slice thickness, 1.2 mm; NEX, 1; bandwidth, 31.25 kHz; acquisition time, 7 min 3 s. Imaging parameters for TOF-MRA were as follows: TR/TE, 23/3.4 ms; flip angle, 18°; FOV, 200 × 200 mm; matrix, 680 × 240; slice thickness, 0.9 mm; NEX, 1; bandwidth, 62.5 kHz; spokes per segment, 288; acquisition time, 5 min 26 s.

DSA was performed for endovascular treatment and postoperative assessment using the ARTIS icono biplane system (Siemens Healthineers, Erlangen, Germany). In addition to conventional DSA images, 3D-DSA (rotational DSA) images and cone beam CT (CBCT) were also obtained.

## Reading experiment

Two radiologists performed two reading experiments separately and in a blinded manner. The first experiment evaluated aneurysm filling and the second evaluated blood flow within the FD. The radiologists did not refer to any clinical information or postoperative course of the patient other than the presented images.

**Fig. 2:**
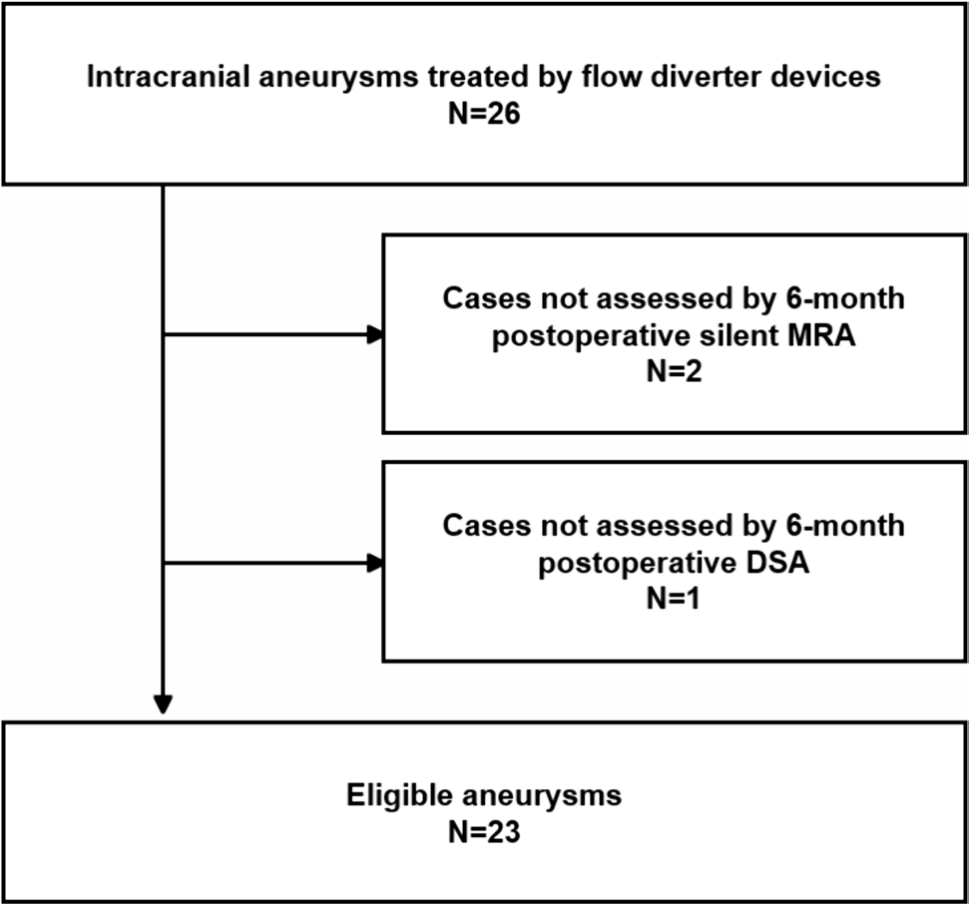
A flowchart of eligible patients assessed using Silent MRA after flow-diverter placement for intracranial aneurysms

We used the OKM scale to evaluate aneurysm filling in DSA. The OKM scale is a treatment efficacy indicator for aneurysms after FD, evaluated using a grade according to the initial degree of filling (A, B, C, and D) and the degree of stasis observed through angiographic phases (arterial, capillary, and venous). We applied the degree of filling (A, B, C, and D) of the OKM scale to MRA as the modified OKM scale and used it to evaluate aneurysm filling. In the modified OKM scale, categories were defined as follows: A (aneurysm filling, > 95%); B (aneurysm filling, 5–95%); C (aneurysm filling, < 5%); or D (aneurysm filling, 0%), similar to the OKM scale (Fig. 2).

One neurosurgeon (with 30 years of experience) performed the evaluation based on the OKM scale for conventional DSA and 3D-DSA images, and two radiologists (with 6–7 years of experience) performed the evaluation based on the modified OKM scale for TOF-MRA and Silent MRA with axial images and maximum intensity projection images. That is to say, the two radiologists evaluated both axial images and maximum intensity projection images for TOF-MRA and Silent MRA. The radiologists visually assessed the post-treatment TOF-MRA and Silent MRA six months after FD placement. No other MRI sequences were provided, but pre-treatment DSA was provided for comparison. If necessary, the two radiologists used pre-treatment CBCT to evaluate TOF-MRA and Silent MRA. The agreement rate between the two radiologists was calculated to demonstrate the reproducibility.

Next, two radiologists evaluated the blood flow within the stent. For this evaluation, the radiologists used post-treatment TOF-MRA, Silent MRA, and DSA six months after FD placement. The degree to which blood flow within the stent could be depicted compared with DSA was scored on a scale of 1–5 (1, almost non-visible; 2, visible but not clearly; 3, moderately visible; 4, clearly visible; 5, visible equivalent to DSA).

The two reading experiments were performed at a one-month interval to minimize the provision of information other than images. First, all the cases of TOF-MRA were evaluated. Then, those of Silent MRA were evaluated. For both TOF-MRA and Silent MRA, the order of MRA cases was randomly shuffled. Because the image difference between TOF-MRA and Silent MRA were apparent for the radiologists, image sequences was not anonymized. That is to say, even if image sequence names were anonymized, the radiologists easily speculated the image sequence names (TOF-MRA or Silent MRA). In cases in which the opinions of the two radiologists differed, a consensus score was determined through discussion.

## Statistical analysis

To evaluate aneurysm filling, we calculated the weighted kappa value for the agreement rate between the OKM scale on DSA and the modified OKM scale on TOF-MRA and Silent MRA [21]. We also calculated the accuracy for the four-class classification (modified OKM scale) and two-class classification (complete occlusion, modified OKM D; incomplete occlusion, modified OKM A, B, and C). The Wilcoxon signed-rank test was performed for statistical analysis of the subjective scores for flow in the stents of both TOF-MRA and Silent MRA. *P* < 0.05 was considered significant. R and EZR were used for statistical analysis [22, 23].

## Results

In the first reading experiment, we evaluated aneurysm filling. The inter-reader agreement rate for the four-class classification (modified OKM scale) was calculated using weighted kappa. The values were 0.793 and 0.803 for TOF-MRA and Silent MRA, respectively, both of which were in substantial agreement. Next, we obtained consensus between the two readers for both TOF-MRA and Silent MRA. The weighted kappa value of the OKM scale between DSA and TOF-MRA for the four-class classification was 0.436, indicating a moderate agreement. The weighted kappa value between DSA and Silent MRA for the four-class classification was 0.943, which was in almost perfect agreement.

Tables 2, 3, 4 show the results of two- and four-class classifications between DSA and MRA. For the accuracy of the four-class classification on the OKM scale (A/B/C/D) was 0.435 for TOF-MRA and 0.870 for Silent MRA. After converting the four-class classification into a two-class classification, the accuracy of the occlusion rate evaluation (complete occlusion/incomplete occlusion) was 0.739 for TOF-MRA and 0.957 for Silent MRA.

**Table 2:** Four-class classification of embolization assessment comparing the OKM scale by DSA with the modified OKM scale by TOF-MRA

		OKM scale by DSA (*n* = 23)			
		A	B	C	D
Modified OKM scale by TOF-MRA (*n* = 23)	A	2	0	0	1
	B	2	1	2	2
	C	0	3	4	2
	D	0	0	1	3

*OKM* O’Kelly-Marotta, *TOF* time of flight

**Table 3:** Four-class classification of embolization assessment comparing OKM scale by DSA with the modified OKM scale by Silent MRA

		OKM scale by DSA (*n* = 23)			
		A	B	C	D
Modified OKM scale by Silent MRA (*n* = 23)	A	3	0	0	0
	B	1	3	0	0
	C	0	1	6	0
	D	0	0	1	8

*OKM* O’Kelly-Marotta

**Table 4:** Two-class classification of embolization assessment for DSA, Silent MRA, and TOF-MRA after flow-diverter stent placement

		DSA (*n* = 23)	
		IO	CO
TOF-MRA (*n* = 23)	IO	14	5
	CO	1	3
Silent MRA (*n* = 23)	IO	14	0
	CO	1	8

we reduced OKM scale to two-class classification: OKM-D for “complete occlusion” and OKM-A/B/C for “incomplete occlusion.”

*OKM* O’Kelly-Marotta,*TOF* time of flight, *CO* complete occlusion, *IO* incomplete occlusion

In the second reading experiment, we evaluated the depiction of blood flow within the stent using a 5-point scale. The mean score for TOF-MRA was 2.43 ± 0.90 and that for Silent MRA was 3.04 ± 1.02. Silent MRA was equal to or better than TOF-MRA in all cases. The Wilcoxon signed-rank test results were *P* < 0.001, indicating that Silent MRA was significantly superior to TOF-MRA in detecting blood flow within the stent.

In addition, signal intensity at the remnant of the aneurysm was evaluated quantitatively. The details are described in Supplementary Material.

## Qualitative evaluation

**Fig. 3:**
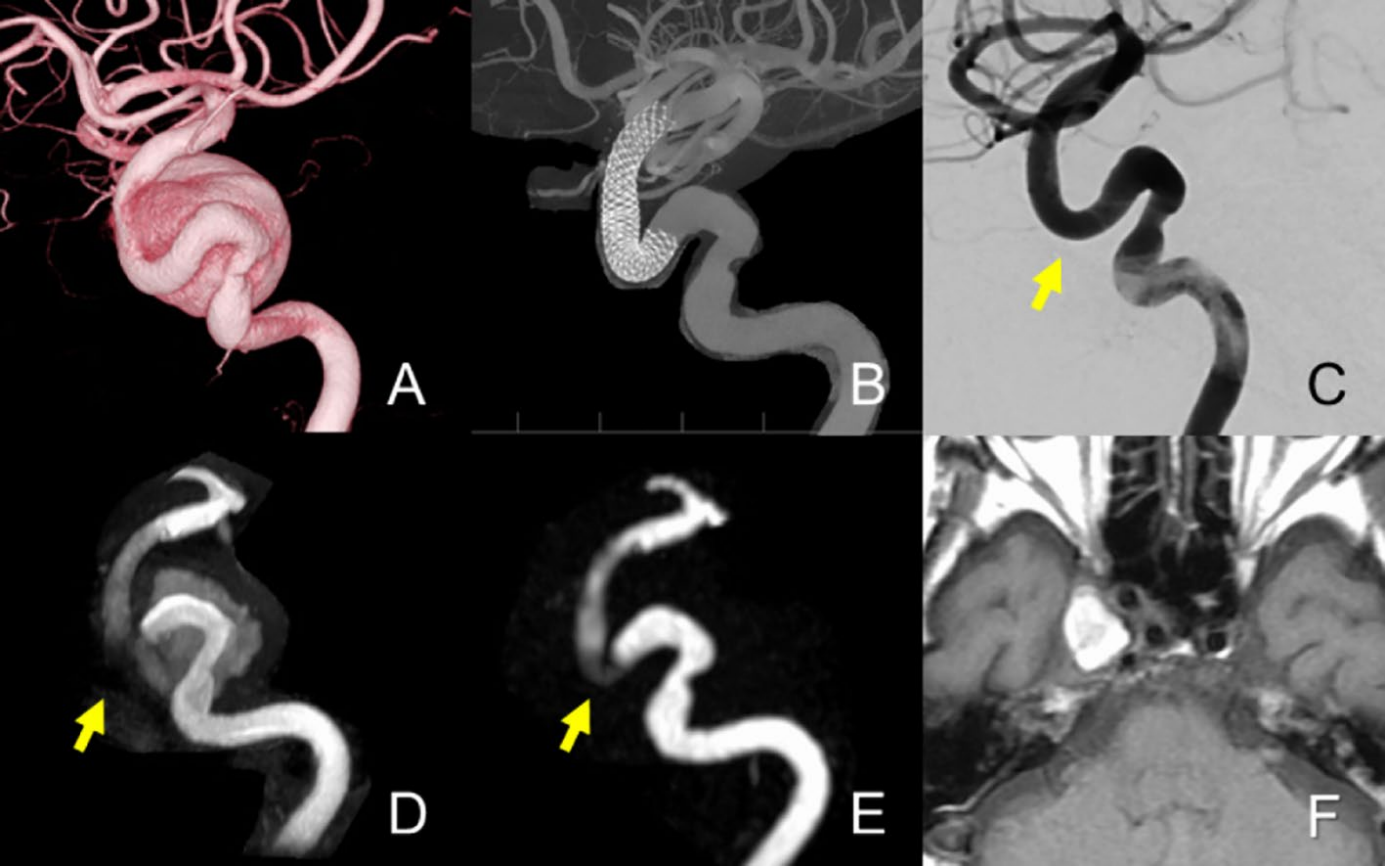
**A** Patient in their 70 s with the right internal carotid artery cavernous segment aneurysm (28 mm). 3D-DSA shows the location of aneurysms using cone beam CT before the treatment. Flow-diverter placement was performed with the pipeline embolization device (PED; 4.5 × 25 mm). (**B**, **C**) The 6-month follow-up maximum intensity projection (MIP) image of cone beam CT and DSA shows complete occlusion (O’Kelly-Marotta [OKM] scale: D) of the aneurysm, and there is no stenosis inside the flow-diverter. **D** TOF-MRA loses the signal in the corner of the Pipeline Flex (arrows) and has a pseudo signal in the aneurysm caused by thrombosis, which has a high signal in the T1-weighted image (**F**). **E** Silent MRA shows a better flow signal in the pipeline flex (arrow) and no intra-aneurysmal signal as DSA. While the OKM scale on DSA was D, those of TOF-MRA and Silent MRA were A and D, respectively. The two radiologists assigned a score 4 after reaching consensus for the Silent MRA, whereas the score for the TOF-MRA were 3

Figure 3 shows a case of a PED. The OKM scale on DSA was D; however, the readers were unable to determine the modified OKM scale as D using TOF-MRA because of the high T1WI signal of the thrombus.

**Fig. 4:**
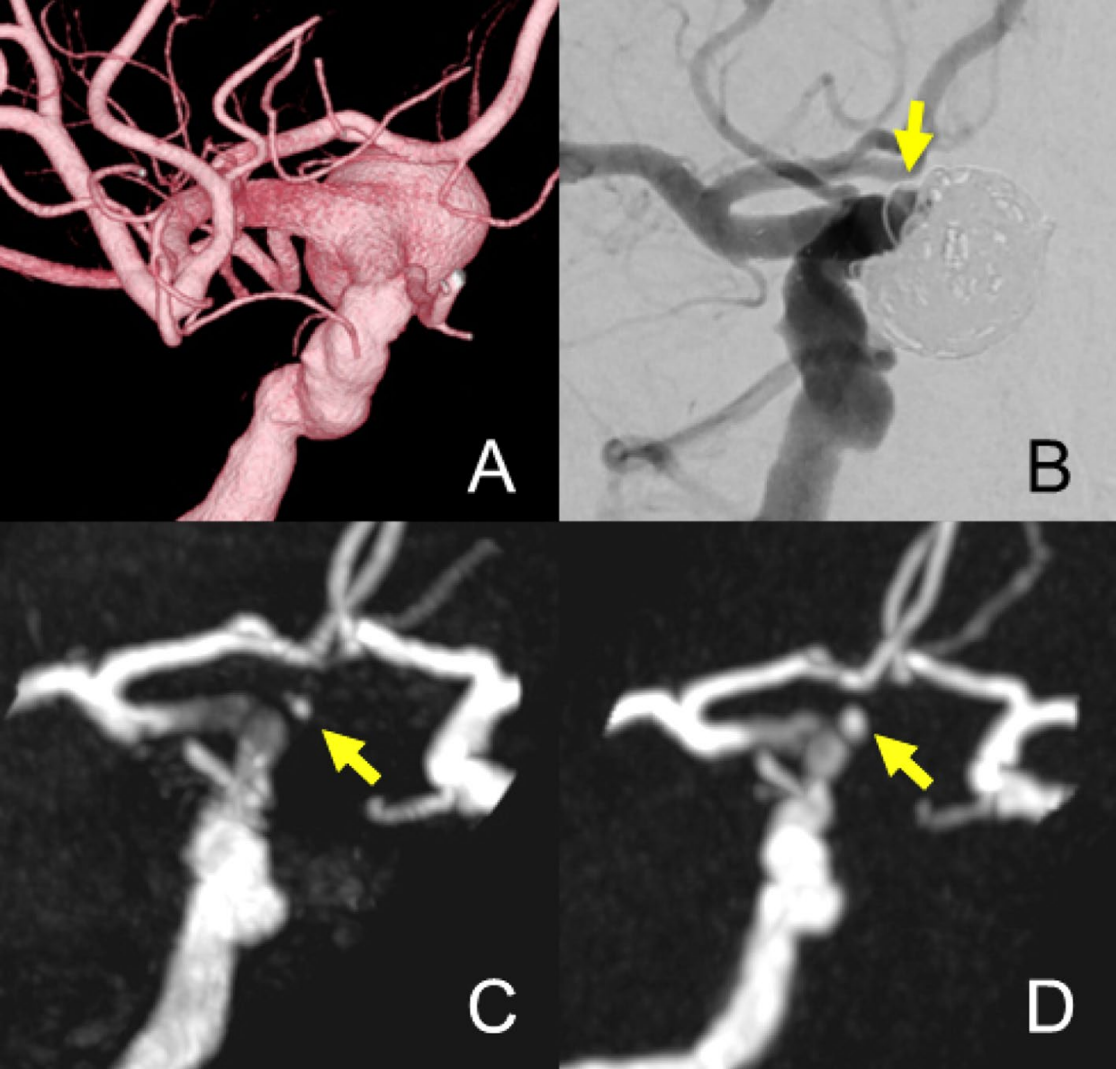
**A** Patient in their 80 s with the right internal carotid artery C2 segment aneurysm (15 mm). 3D-DSA shows the location of aneurysms before the treatment. Flow-diverter placement was performed with FRED (5.0 × 21 mm) and coils. **B** The 6-month follow-up DSA shows neck remnant of the aneurysm, and there is no stenosis inside the flow-diverter. (**C**, **D)**, both TOF-MRA and Silent MRA show neck remnants of an aneurysm. The OKM scale in DSA was C, and those of TOF-MRA and Silent MRA were C as well. The score after consensus of the Silent MRA by 2 radiologists was 4, and that of the TOF-MRA was 3

Figure 4 shows a case treated with FRED and additional coils. Silent MRA showed a better depiction of blood flow within the aneurysm and stent than TOF-MRA. Both TOF-MRA and Silent MRA show neck remnant of the aneurysm.

**Fig. 5:**
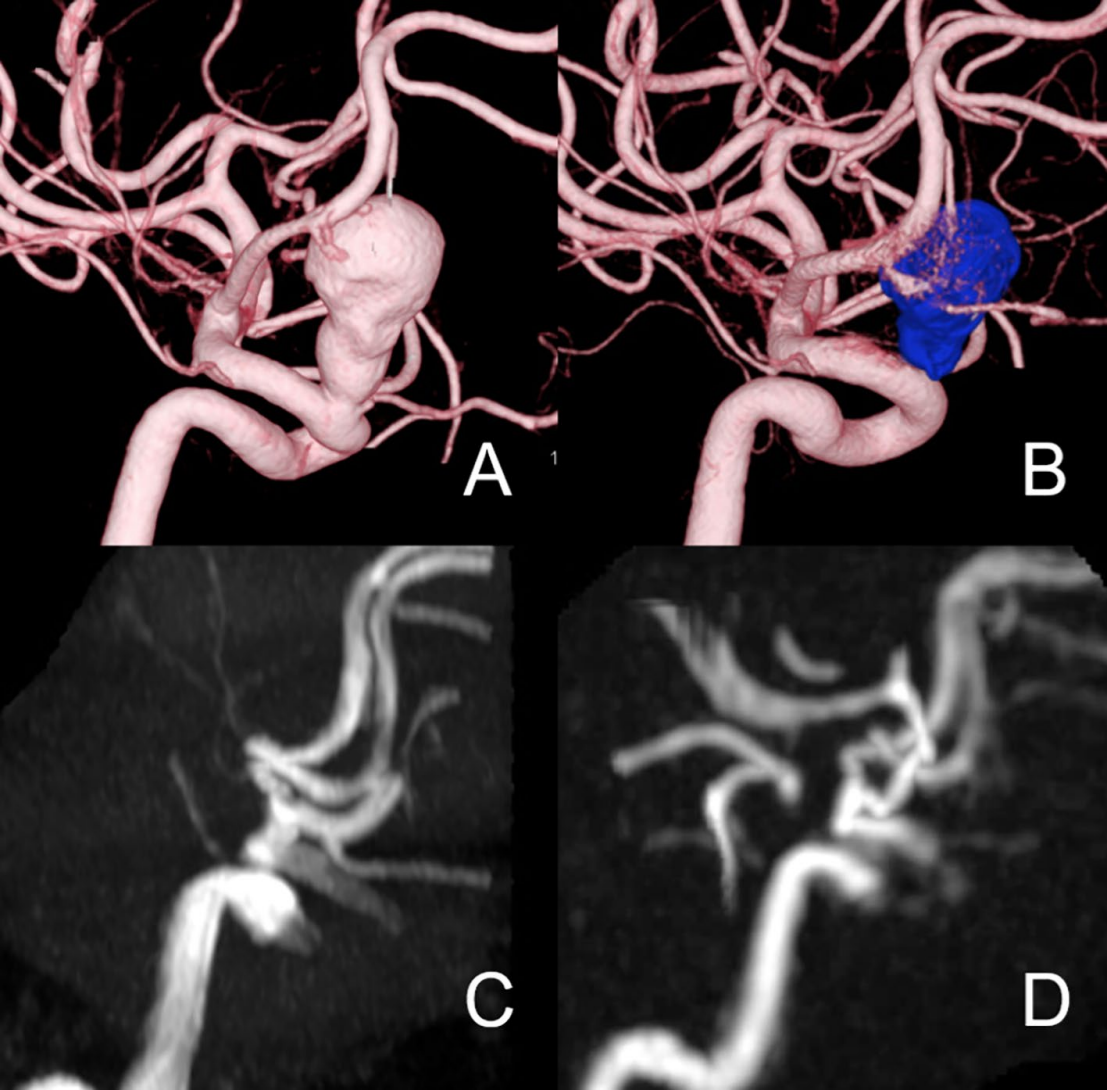
**A** Patient in their 40 s with the left internal carotid artery C2 segment aneurysm (16.5 mm). 3D-digital subtraction angiography (3D-DSA) shows the location of aneurysms before the treatment. Flow-diverter placement was performed with the PED (3.75 × 18 mm) and additional coil. **B** The 6-month follow-up 3D-DSA show complete occlusion (OKM scale: D) of the aneurysm, and there is no stenosis inside the Flow-diverter. **C** 6-month follow up TOF-MRA. **D** 6-month follow up Silent MRA. The OKM scale in DSA was D, and those of TOF-MRA and Silent MRA were both D. The score after consensus of the Silent MRA by 2 radiologists was 3, and that of the TOF-MRA was 2

**Fig. 6:**
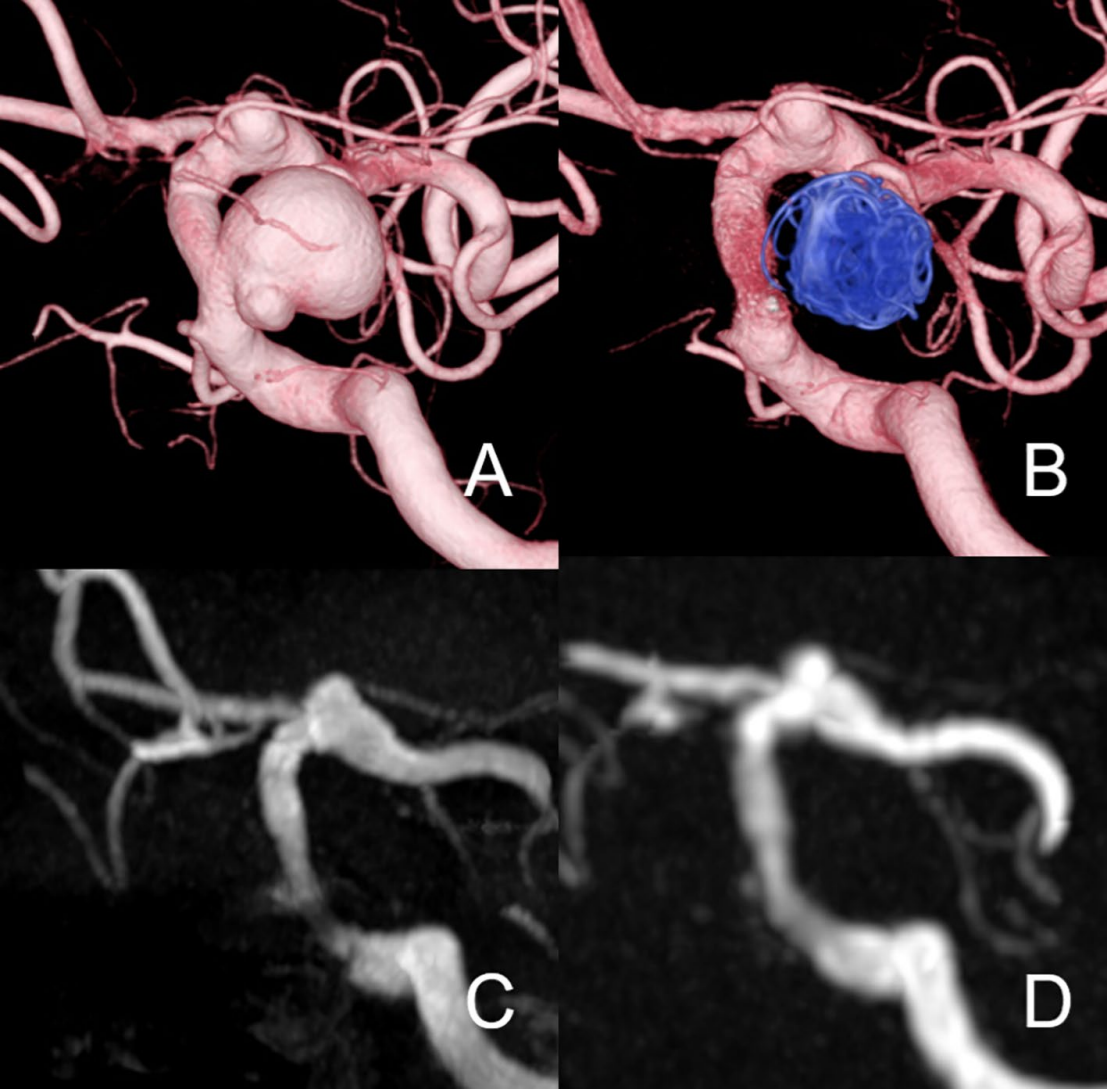
**A** Patient in their 70 s with the left internal carotid artery C2 segment aneurysm (14 mm). 3D-digital subtraction angiography (3D-DSA) shows the location of aneurysms before the treatment. Flow-diverter placement was performed with the PED (4.75 × 20 mm) and additional coil. **B** The 6-month follow-up 3D-DSA show complete occlusion (OKM scale: D) of the aneurysm, and there is no stenosis inside the Flow-diverter. **C** 6-month follow up TOF-MRA. **D** 6-month follow up Silent MRA. The OKM scale in DSA was D, and those of TOF-MRA and Silent MRA were both D. The score after consensus of the Silent MRA by 2 radiologists was 4, and that of the TOF-MRA was 3

**Fig. 7:**
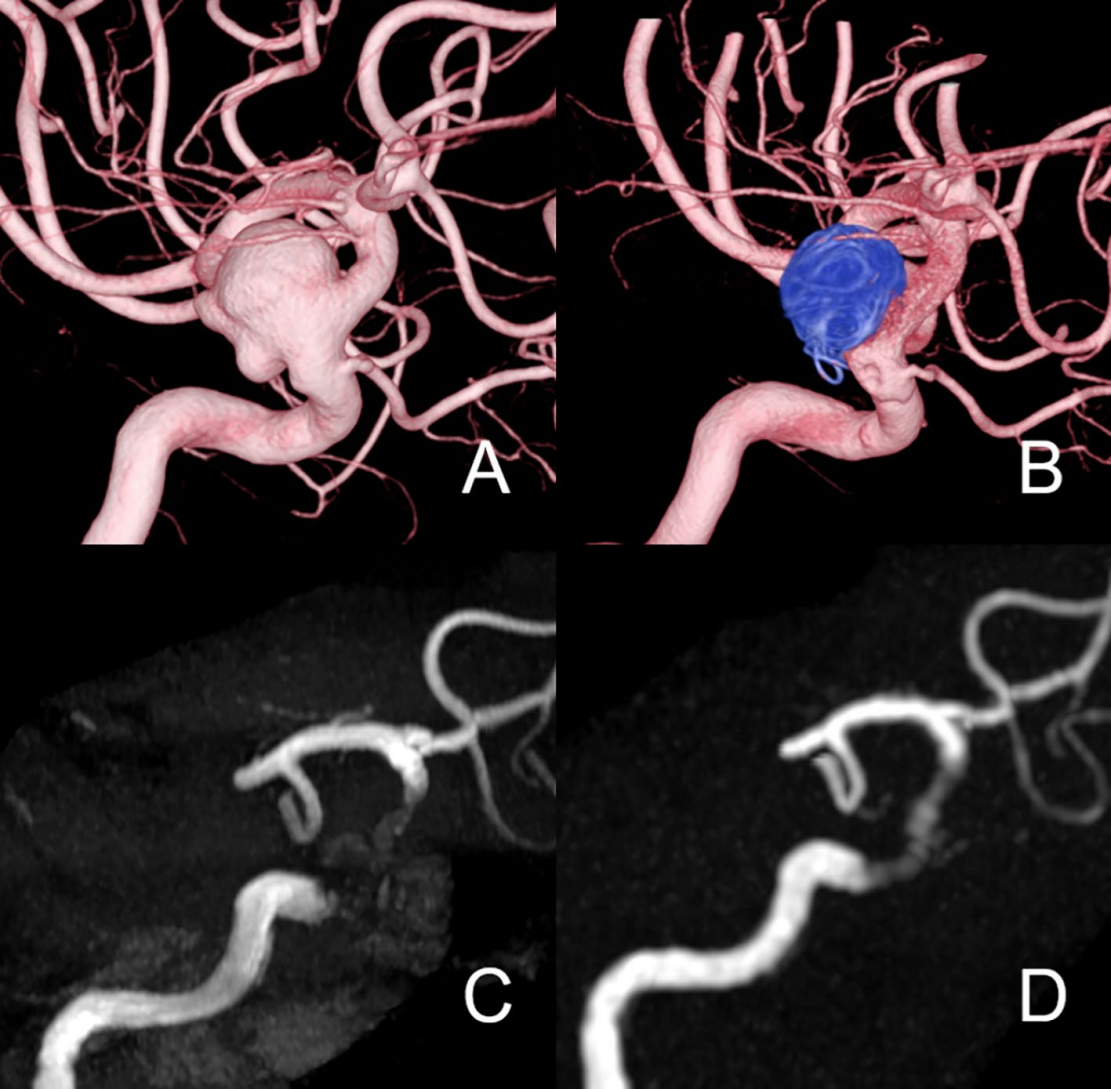
**A** Patient in their 70 s with the right internal carotid artery C2 segment aneurysm (13 mm). 3D-digital subtraction angiography (3D-DSA) shows the location of aneurysms before the treatment. Flow-diverter placement was performed with the PED (4.50 × 16 mm) and additional coil. **B** The 6-month follow-up 3D-DSA show complete occlusion (OKM scale: D) of the aneurysm, and there is no stenosis inside the Flow-diverter. **C** 6-month follow up TOF-MRA. **D** 6-month follow up Silent MRA. The OKM scale in DSA was D, and those of TOF-MRA and Silent MRA were both D. The score after consensus of the Silent MRA by 2 radiologists was 4, and that of the TOF-MRA was 3

Figures 5, 6, 7 present the MRA and DSA results for the other three cases treated with FD and additional coils. Even in patients treated with coils, within the aneurysm and stent, Silent MRA provided a blood flow depiction more similar to DSA than that of TOF-MRA.

## Discussion

In our study, Silent MRA was superior to TOF-MRA for depicting aneurysm filling and in-stent blood flow after FD placement. In particular, when assessing aneurysm filling using the modified OKM scale, an almost perfect agreement was observed between Silent MRA and DSA, demonstrating that the diagnostic accuracy of Silent MRA was sufficiently high, which highlights its clinical utility. This is likely because Silent MRA reduces metal artifacts and can discriminate between high T1WI signals from the thrombus and high signals related to blood flow.

Oishi et al. studied 78 cases of FD placement and found Silent MRA to be superior compared with TOF-MRA in depicting stent intraluminal blood flow and aneurysm filling [18]. Aneurysm filling was evaluated using a two-class classification for complete and incomplete occlusions, and the accuracies of Silent MRA and TOF-MRA were 91.0% and 80.8%, respectively. In our study, we evaluated aneurysm filling using a two-class classification, as in Oishi et al., and a four-class classification using the modified OKM scale. In our study, the accuracies of Silent MRA and TOF-MRA for the four-class classification were 87.0% and 43.5%, respectively, whereas they were 95.7% and 73.9% for the two-class classification, respectively. We used the four-class classification with the modified OKM scale for evaluation because some studies and surgeons regard the OKM scale C + D as satisfactory occlusion [24–26]. The modified OKM scale provided a more precise assessment of aneurysm filling than the two-class classification, making the difference in diagnostic ability between Silent MRA and TOF-MRA even more apparent.

In the study by Oishi et al., it took approximately 12 min to acquire Silent MRA images, whereas in our study, it took approximately 7 min. We attempted to accurately compare Silent MRA and TOF-MRA by reducing the imaging time compared with Oishi et al. Therefore, we believe that our Silent MRA protocol for acquiring images in shorter scan time would certainly be advantageous in clinical practice.

By comparing the MRI scanners and scan protocols between our study and that of Oishi et al., it has been speculated that the time difference might be caused by the difference in the MRI scanners. Although the value of spokes per segment might affect the scan time, the value of spokes per segment is not described in the paper of Oishi et al. Therefore, it is impossible to compare the value of spokes per segment between our study and that by Oishi et al.

The advantages and disadvantages of DSA, CTA, TOF-MRA, and Silent MRA are summarized in Supplementary Table. Although DSA is the gold standard, it requires contrast agents and radiation exposure, and is associated with a certain percentage of complications [27] compared to CTA or MRA. Patients can be followed up for several years after FD placement. Therefore, a less-invasive testing method is required. CTA is less invasive than DSA, but still requires the use of contrast agents and radiation exposure. In contrast, non-contrast MRA (TOF-MRA and Silent MRA) is non-invasive and does not require contrast agents, making it suitable for the long-term follow-up of FD placement. Based on the results of our study, Silent MRA may be more useful than TOF-MRA for the follow-up after FD placement.

Follow-up MRA is especially important in patients treated with both FD and additional coils [28] because the presence of coil artifacts renders CTA imaging unclear. Therefore, in patients treated with both FD and additional coils, the benefit of Silent MRA may be higher than in patients treated with FD alone.

FRED has been used for FD placement recently [29]. Compared to the visibility of blood flow within the stent using FD placement with the pipeline stent, those with FRED might show a slightly better tendency. This difference might be attributed to the metal components of the stent, which can result in varying degrees of metal artifacts. However, the number of patients treated with FRED in the present study was small. Therefore, future research should include a more comprehensive evaluation of patients treated with FRED.

FD placement indications now include posterior circulation arteries such as the vertebral artery and basilar artery aneurysms [30, 31]. However, there are no studies on the use of Silent MRA in these cases; therefore, further investigation of the posterior circulation arteries is needed. Silent MRA is expected to be useful for assessing aneurysm filling in these cases. However, in the vertebral and basilar arteries, the assessment of in-stent blood flow is expected to be more challenging than that in the ICA because the luminal diameter inside the stent is even smaller.

This study has several limitations. First, the study sample size is small. This is because giant internal carotid artery aneurysms are relatively rare and FD has only recently become an insurance-covered treatment option. In particular, the number of cases with the FRED was even smaller than that with the PED. Therefore, larger studies specifically focused on FRED in the future are required. Second, the assessment of intraluminal blood flow within the stent using Silent MRA may not be high enough to diagnose stenosis within the stent definitively. Third, there were no cases of in-stent stenosis at the six-month follow-up DSA [4, 32, 33]. Therefore, the diagnosis of in-stent stenosis was not evaluated in this study. Considering the clinical utility of Silent MRA in-stent imaging, it would be better to compare the diagnoses of DSA, Silent MRA, and TOF-MRA, preferably for the presence of in-stent stenosis.

## Conclusions

We compared the modified OKM scale on MRA and the original OKM scale in DSA to evaluate the usefulness of Silent MRA and TOF-MRA for patients with aneurysms after FD placement. Silent MRA showed a high degree of agreement in aneurysm filling with DSA compared with TOF-MRA. Therefore, Silent MRA is clinically useful for the follow-up of patients after FD placement.

## Supplementary Information

Below is the link to the electronic supplementary material.

Supplementary file1 (DOCX 55 KB)

## Abbreviations

TOF: Time of flight
FD: Flow-diverter
PED: Pipeline embolization device
FRED: Flow-direction endoluminal device
OKM: O’Kelly-Marotta
CBCT: Cone beam CT
