# Supplementary material for: Predicting the O’Kelly-Marotta scale score after flow-diverter stent placement using silent MRA
Source: Jpn J Radiol. 2024 Aug 29;42(12):1403–12. doi: 10.1007/s11604-024-01632-1 (PMC11588759; doi:10.1007/s11604-024-01632-1)
Supplement: Supplementary file 1 — Supplementary file1 (DOCX 55 KB) [file 11604_2024_1632_MOESM1_ESM.docx]

**Supplementary Material**

**Supplementary Document.**

**Material and Method**

For the aneurysm whose OKM scale was A, B, or C, the signal intensity of TOF-MRA and Silent MRA was evaluated. For this evaluation, one radiologist placed a region of interest (ROI) at the remnant of the aneurysm and the main artery without the aneurysm (contralateral internal carotid artery or basilar artery). Then, the average of the signal intensity at two ROIs was obtained. Finally, the ratio of the averaged signal intensity at the remnant to that at the main artery was calculated. As this ratio is higher, the visibility of the remnant is higher.

**Results**

There were 12 aneurysms whose OKM scale was A, B, or C. The ratio of signal intensity at the remnant was 0.8286±0.2452 for silent MRA; 0.7606±0.1914 for TOF MRA. Supplementary Figure 1 shows the box plot for the ratio of signal intensity. Because the ratio on Silent MRA was higher than that on TOF-MRA, the visibility of the remnant might be higher on Silent MRA than that on TOF-MRA.


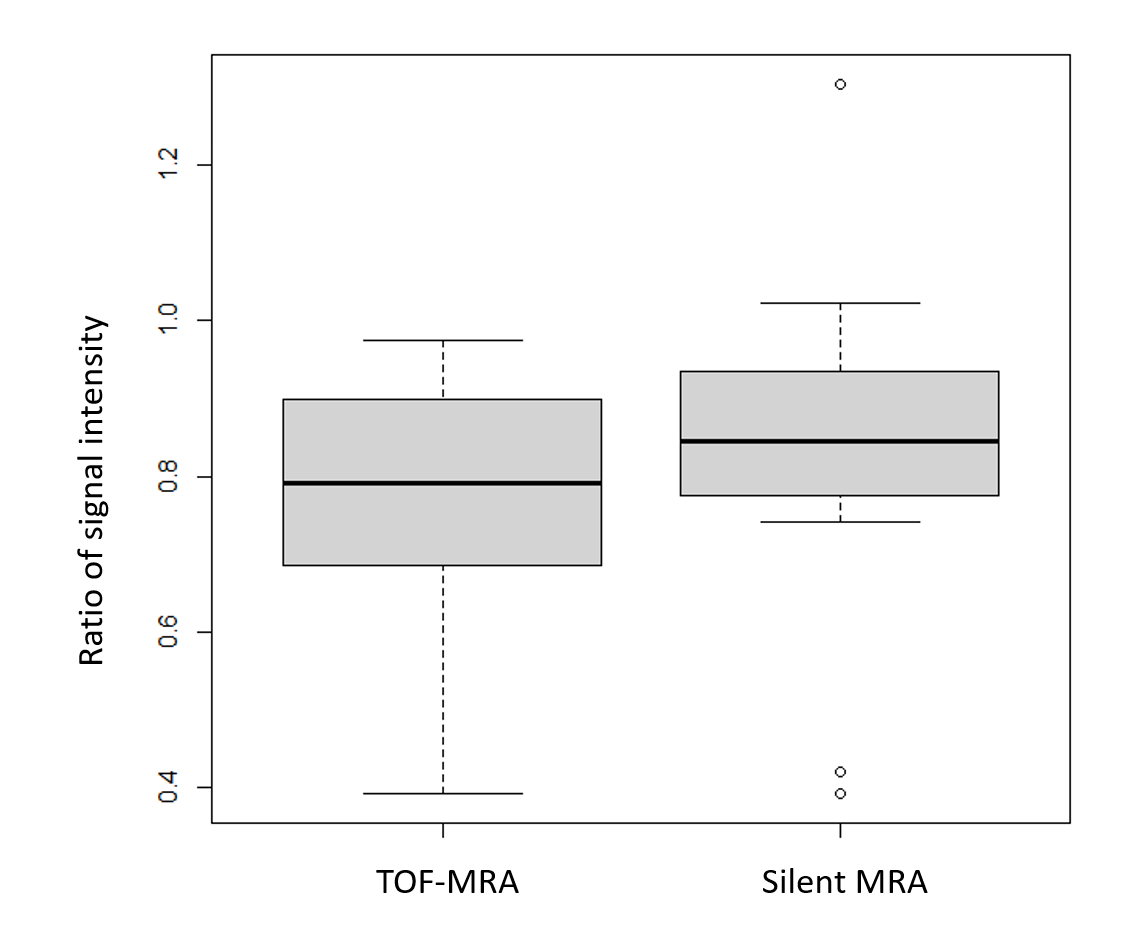


**Supplementary Figure.** Box plot for the ratio of signal intensity.

**Supplementary Table.** Summary of imaging modalities for intracranial aneurysm

| Item | DSA | CTA | TOF-MRA | Silent MRA |
| --- | --- | --- | --- | --- |
| Evaluation for aneurysm filling | Excellent | Excellent | Good or Fair | Excellent |
| Visualization in stent | Excellent | Excellent | Good or Fair | Good or Fair |
| Visualization around coiling | Excellent | Poor | Good or Fair | Excellent |
| Radiation exposure | High | Relatively high | None | None |
| Contrast media | ＋ | ＋ | – | – |
| Time required | Hospitalization | A few minutes | Approximately 30 min for whole MRI | Approximately 30 min for whole MRI |
